# The impact of considering land intensification and updated data on biofuels land use change and emissions estimates

**DOI:** 10.1186/s13068-017-0877-y

**Published:** 2017-07-20

**Authors:** Farzad Taheripour, Xin Zhao, Wallace E. Tyner

**Affiliations:** Department of Agricultural Economics, Purdue University, West Lafayette, USA

**Keywords:** Land use change, Biofuel emissions, Intensive versus extensive margin, GTAP model and database

## Abstract

**Background:**

The GTAP model has been used to estimate biofuel policy induced land use changes and consequent GHG emissions for more than a decade. This paper reviews the history of the model and database modifications and improvements that have occurred over that period. In particular, the paper covers in greater detail the move from the 2004 to the 2011 database, and the inclusion of cropland intensification in the modeling structure.

**Results:**

The results show that all the changes in the global economy and agricultural sectors cause biofuels induced land use changes and associated emissions can be quite different using the 2011 database versus 2004. The results also demonstrate the importance of including land intensification in the analysis. The previous versions of GTAP and other similar models assumed that changes in harvested area equal changes in cropland area. However, FAO data demonstrate that it is not correct for several important world regions. The model now includes land intensification, and the resulting land use changes and emission values are lower as would be expected.

**Conclusions:**

Dedicated energy crops are not similar to the first generation feedstocks in the sense that they do not generate the level of market-mediated responses which we have seen in the first-generation feedstocks. The major market-mediated responses are reduced consumption, crop switching, changes in trade, changes in intensification, and forest or pasture conversion. These largely do not apply to dedicated energy corps. The land use emissions for cellulosic feedstocks depend on what we assume in the emissions factor model regarding soil carbon gained or lost in converting land to these feedstocks. We examined this important point for producing bio-gasoline from miscanthus. Much of the literature suggests miscanthus actually sequesters carbon, if grown on the existing active cropland or degraded land. We provide some illustrative estimates for possible assumptions. Finally, it is important to note the importance of the new results for the regulatory process. The current California Air Resources Board carbon scores for corn ethanol and soy biodiesel are 19.8 and 29.1, respectively (done with a model version that includes irrigation). The new model and database carbon scores are 12 and 18, respectively, for corn ethanol and soy biodiesel. Thus, the current estimates values are substantially less than the values currently being used for regulatory purposes.

## Background

The GTAP-BIO model has been developed and frequently improved and updated to evaluate biofuels induced land use changes and their consequent emissions [1–7]. The modifications made in this model can be divided into three groups: modifications and updates in the GTAP-BIO database; changes in model parameters; and improvements in the modeling structure. This paper briefly reviews these changes, introduces a set of new modifications into the model and its database, and examines induced land use emissions for several biofuel pathways using the new model and its database.

The previous version of this model uses an old databases (GTAP database version 7) which represents the world economy in 2004. During the past decade, the global economy has changed considerably. In particular, since 2004, major changes occurred in the agricultural and biofuel markets. Recently, a new version of the GTAP database (version 9) which represents the world economy in 2011 has been published. However, as usual, this standard database does not explicitly represent production and consumption of biofuels. We have added biofuels (including traditional biofuels and several advanced cellulosic biofuels) into this database to take the advantages of the newer databases. This allows us to examine the economic and land use consequences of the first- and second-generation biofuels using the updated database.

Several recent publications [8–15] have shown that that land intensification in crop production (in terms of expansion in multiple cropping and/or returning unused cropland to crop production) has increased in several regions across the world. Typically, economic models, including GTAP-BIO, ignore this kind of intensification. Recently, we improved the GTAP-BIO model to take into account land intensification in crop production. We use this model in combination with the new database mentioned above to assess the land use impacts of several biofuel pathways. We compare the results of the new simulations with their corresponding results obtained from the older versions.

## Methods

### GTAP-BIO database version 9

The standard GTAP databases do not include production, consumption, and trade of biofuels. Taheripour et al. [16] introduced the first generation of biofuels (including grain ethanol, sugarcane ethanol, and biodiesel) into the GTAP standard database version 6, which represented the world economy in 2001 [17]. The early versions of the GTAP-BIO model were built on this database and used in several applications and policy analyses [3, 4, 18–21]. The California Air Resources Board (CARB) developed its first set of ILUC values using this database and early versions of the model [22]. The Argonne National Lab also used the results of this model in developing the early versions of the life cycle analyses (LCA) of biofuels [21, 23].

When the standard GTAP database version 7, which represented the world economy in 2004 was released [24], Taheripour and Tyner [25] introduced first- and second-generation biofuels into this database. Several alternative aggregations of this database have been developed and used in various studies to evaluate the economic and land use impacts of biofuel production and polices [26–31]. CARB has used this database to develop its final ILUC values [32, 33], and Argonne National Lab also used the outcomes obtained from this database in its more recent LCA analyses.

The GTAP-BIO 2004 database in comparison to its 2001 version had several advantages including but not limited to: (1) providing data on cropland pasture for the US and Brazil; (2) disaggregating oilseeds into soybeans, rapeseed, palm, and other oilseeds; (3) disaggregating coarse grains into sorghum and other coarse grains; (4) introducing cellulosic crops and corn stover collection as new activities into the database; (5) disaggregating vegetable oil industry into soybean oil, rapeseed oil, palm oil, and other vegetable oils and fats and their corresponding meal products; (6) dividing the standard food industry of GTAP into two distinct food and feed industries; and (7) covering a wide range of biofuels including ethanol produced from grains, ethanol produced from sugar crops, four types of biodiesel produced from soybean oil, rapeseed oil, palm oil, and other oils and fats, three types of cellulosic ethanol produced form corn stover, switchgrass, and miscanthus and three types of drop-in cellulosic biofuels produced from the corn stover, switchgrass, and miscanthus.

The GTAP-BIO 2004 database with all of the above advantages is now out-of-date. During the past decade, the global economy has changed significantly with major consequences for agricultural and energy markets including biofuels. On one hand, demand for agricultural products has increased across the world at different rates due to growths in income and population. Expansion in biofuel production due to public policies has contributed to the expansion in demand for agricultural products in some regions and at the global scale, as well. On the other hand, the agricultural sector has evolved considerably across the world: crop production and its geographical distribution have changed, the mix of crops produced in most countries has changed, crop yields have improved due to technological progress in many regions, crop production has been negatively affected in some regions due to severe climate conditions, and international trade in agricultural products has changed. Major changes occurred in the livestock industry, as well: demand for meat and meat products has shifted from red meat towards white meat, more biofuels by-products and meals were used in animal feed rations, and land intensification has been extended in the livestock industry. The biofuel industry has grown rapidly across the world and, in particular, in US, Brazil, and EU. Biofuel producers now operate more efficiently than before. Unlike the early 2000s, the biofuel industry is now a mature industry which operates without government subsidies. However, they still benefit from biofuel mandates. The 2004 database misses all these changes and many other changes which occurred in the global economy. Therefore, it becomes necessary to update the GTAP-BIO database.

To accomplish this task, following our earlier work in this area [16, 25, 34], we explicitly introduced biofuels into the latest publicly released version (V9) of the standard GTAP database which represents the world economy in 2011 [35]. This means is that all the steps that we followed to introduce biofuels into the 2001 and 2004 databases had to be repeated for the 2011 GTAP database but using 2011 data for all the biofuels components. Thus, production, consumption, trade, prices, and co-products had to be introduced into the 2011 database. The full description of this task is reported in [36]. Here, we explain the main important aspects of this task.

#### Data collection

Production and consumption of biofuels for 2011 are taken from the US Energy Information Administration (EIA) website (http://www.eia.gov). The EIA provides data on ethanol and biodiesel produced across the world by country. Harvested area, crop produced, area of forest, pasture, and cropland for 2011 are obtained from the FAOSTAT database http://faostat3.fao.org/home\E; for details, see [37]. Data on vegetable oils and meals produced, consumed, and traded in 2011 were collected by country from the world oil database [38] and used to split the GTAP vegetable oil sector into different types of vegetable oils and meals.

#### Introducing new non-biofuel sectors into the standard database

As mentioned above in our earlier work [16, 25, 34], we developed a process to further disaggregate coarse grains, oilseeds, vegetable oils, and food sectors of the GTAP original database to additional new sectors to support various biofuel pathways and their links with the agricultural, livestock, food, and feed industries. Using the collected data mentioned in “Data collection” section, we repeated that process for the 2011 database.

In addition, unlike the earlier versions of the GTAP-BIO databases, a blend sector was added to the database to represent a new industry which blends biofuels with traditional fuels. The earlier versions of this database assumed that biofuels are directly used by the refinery sector (as an additive to the traditional fuels) or consumed by households (as substitutes for the traditional fuels). The new blend sector takes the traditional fuels used in transportation and blends them with biofuels. This sector supplies the blended fuels to the transportation sectors and final users.

#### Introducing biofuel sectors into the standard database

In our earlier work [16, 25, 34], a process was also designed and implemented to introduce biofuels into a standard GTAP database. We followed and improved that process to introduce biofuels into the GTAP database version 9. This process first determines the original GTAP sectors which biofuels are embedded. Then, data were obtained on monetary values of biofuels produced by country; a proper cost structure for each biofuel pathway; users of biofuels; and feedstock for each biofuel. Finally, it uses these data items and a set of programs to introduce biofuels into the database. As an example, in the standard GTAP database, the US corn ethanol is imbedded in the food sector. Therefore, this sector was divided into food and ethanol sectors. To accomplish this task, we needed to evaluate monetary values of corn ethanol and its by-product (DDGS) produced in the US at 2011 prices. We also needed to determine the cost structure of this industry in the US in 2011, as well. This cost structure should represent the shares of various inputs (including intermediate inputs and primary factors of production) used by the ethanol industry in its total costs in 2011. For the case of US corn ethanol, which represents a well-established industry in 2011, these data items should match with national level information. Hence, as mentioned in the previous section, we collected data from trusted sources to prepare required data for all types of the first generation of biofuels produced across the world in 2011. For the second generation of biofuels (e.g., ethanol produced from switchgrass or miscanthus) which are not produced at commercial level, we rely on the literature to determine their production costs and also their cost structures. For these biofuels, we also need to follow the literature to define new sectors (e.g., miscanthus or switchgrass) and their cost structures to include their feedstock at 2011 prices.

After preparing this information, we used a set of codes and the SplitCom program [39] to insert biofuels into the national input–output tables of the standard database. The SplitCom program allows users to split a particular sector into two or more sectors while maintaining the national SAM tables in balance. To split a particular sector, the program takes the original database (including regional SAM tables) and some additional external data items and then runs the split process. In general, in each split process, the additional external data items are: (1) the name of original sector; (2) the name of new sectors; (3) the cost structure of new sectors; (4) users of the new sectors; (5) share of each user in each new product; and (6) trade flows of new products. See these references for more details [16, 25, 34, 36].

#### Other important data modifications

In addition to the above modifications, we made several adjustments in the standard GTAP database to match with real-world observations. The major adjustments are:

- Production and sales of US coarse grains are adjusted according to the USDA data. The modified GTAP-BIO US input–output table shows that 11.3, 26.8, and 61.9% of corn used by livestock industry are consumed by dairy, ruminant, and non-ruminant subsectors, respectively. The corresponding original GTAP figures are about 48, 7, and 45%. We altered the original GTAP figures to match with the USDA data.
- The standard GTAP database underestimates the monetary value of vegetable oils and their meals produced in the US. This is fixed using the world oil database [38]. According to this database which reports vegetable oils and meals produced across the world and using a set of price data for these products obtained from the FAOSTATA, we estimated that the US vegetable oil industry produced about $36.5 billion in 2011. The corresponding GTAP figure was about $25 billion.
- The monetary values of vegetable oils used in non-food uses presented in the input–output tables of some countries were smaller than the monetary values of vegetable oils needed to support their biodiesel production. The input–output tables of these countries were properly modified to solve these inconsistencies.
- Cropland pasture data were added for Canada [39], and proper changes were made in the input–output table of this country. Cropland pasture was updated for the US and Brazil according to the existing data for 2011.

The GTAPADJUST program developed by Horridge [40] and several programs developed by the authors were used to carry out the above changes and adjustments. The GTAPADJUST program allows users to modify elements of the SAM tables while maintaining required balances.

In conclusion, the GTAP-BIO databases for 2004 and 2011 represent the same regional and sectoral aggregation schemes, except for the blend sector which was added to the 2011 database. While these two databases represent the same aggregation schemes, they represent entirely different data content. Finally, it is important to note that a GTAP-BIO database including cellulosic biofuels is labeled GTAP-BIO-ADV. The GTAP-BIO and GTAP-BIO-ADV versions for each year represent the same data contents, but the latter represents the second-generation biofuel pathways with very small production levels.

#### Database comparison

Here, we briefly compare the new GTAP-BIO database which represents the world economy in 2011 with the 2004 version. See [36] for the full comparison of these two databases. Note that in CGE models, the data for the base year represent all economic data for that year, and, in some circumstances, because of annual variability, the base year may not be completely representative of trends. The impacts of this issue normally are not large, but it is an issue for all CGE models.

***Expansion in biofuel production*** Total biofuel production (including ethanol and biodiesel) has rapidly increased from 8.4 billion gallons (BGs) in 2004 to 29 BGs in 2011 at the global scale, a tremendous growth of 19.4% per year over this time period. In 2004, Brazil, US, and EU were the main biofuel producers. In this year, they were producing about 4, 3.4, and 0.7 BGs biofuels (manly ethanol), respectively. In 2011, about 22.9 BGs of ethanol and 6.2 BGs of biodiesel were produced across the world. The largest ethanol producers including US, Brazil, and EU produced 13.9, 6, and 1.1 BGs of ethanol in 2011. The next three largest ethanol producers were China (with 0.6 BGs), Canada (0.5 BGs), and South America (0.2 BGs). The largest biodiesel producers including EU, US, and South America produced 2.7, 1, and 0.9 BGs of biodiesel in 2011. The next three largest biodiesel producers were Brazil (with 0.7 BGs), Malaysia and Indonesia (0.3 BGs), and South East Asia (0.2 BGs).

***Economy-wide comparison*** Many changes occurred in the global economy. Population increased by about 550.4 million across the world between 2004 and 2011. Major changes occurred in sub-Saharan Africa (144.2 million or 19.6%), India (by 134 million or 12.3%), and Middle East and North Africa (48.6 million or 14.2%). In most developed countries and regions, population has been increased slightly or decreased.

In 2004, EU, US, and Japan had the largest shares in the global production of goods and services (measured with GDP) with 31.5, 28.5, and 11.4% shares, respectively. In 2011, the shares of these regions dropped to 24.6%, 21.7, and 8.3%. Instead, the share of China from global productions of goods and services has increased from 4.6% in 2004 to 10.6% in 2011. As a measure of income, GDP per capital at current prices has increased all across the world in 2004–11. Large changes occurred in China (301%), Brazil (274%), and Russia (236%).

The share of consumption and investment in GDP in 2004 and 2011 are not very different in many regions. However, some regions like China, India, East Asia, Malaysia–Indonesia, and Russia allocated larger shares of their GDP to investment and spend less on consumption in 2011 compared with 2004.

Between 2004 and 2011, in several regions across the world, the shares of agricultural, processed food and feed, biofuels, and energy sectors in GDP increased, but the total share of other goods and services decreased. Some countries experienced differently. For instance, the agricultural share in total output declined in some countries such as Brazil, China, and India. In these countries, agricultural activities experienced rapid growths, but their growth rates were smaller than the growth rates of other economic activities.

At the national level, the shares of domestic and export uses in total value of output of each region have not significantly changed. However, at commodity level, important changes occurred. For example, consider a few examples from the US economy. In 2004, the US exported 32% of its coarse grains to other countries. This figure was about 19% in 2011. That is basically is due to the expansion in domestic use of corn for ethanol production. On the other hand, the US exports of DDGS have increased from 1 million metric tons in 2004 to about 8 million metric tons in 2011. During this time period, the share of exports in total output of soybeans increased from 44 to 53%. As another example, the share of domestic use in total energy produced in the US decreased from 97% in 2004 to 91% in 2011.

The regional GTAP input–output tables represent the cost structure of sectors/industries in each region. The cost structures of the well-established sectors have not significantly changed. However, changes are large for the ethanol and biodiesel sectors. These industries were relatively new in 2004 with large shares for capital and smaller shares for feedstocks. In 2011, these industries became more mature and well established with lower shares for capital and higher shares for feedstock. For example, the share of capital in total costs of ethanol sector dropped from 52.2% in 2004 to 18.5% in 2011. That reflects the fact that emerging sectors use more capital at the early stages of their development paths. When well established, the share of capital usually drops, but the share of intermediate inputs goes up. For example, the share of non-energy intermediate inputs (mainly corn) in total costs of ethanol sector increased from 38.3% in 2004 to 76.1% in 2011. This difference is also due to the higher corn price in 2011 compared with 2004. Notice that the price of corn was exceptionally high in 2011, and therefore, the share of this input in total cost of ethanol was slightly higher in this year. This share has been around 65 to 75% in recent years.

***Biophysical data*** The GTAP-BIO database includes data on land cover, harvested area, and crop production by region. It also represents cropland pasture in a few counties. Here, we examine changes in these variables between 2004 and 2011.

*Land cover* At the global scale, areas of forest and cropland increased by 7.8 and 17.5 million hectares, respectively, while area of pasture decreased by 41.7 million hectares. This means that at the global scale, the livestock industry in 2011 is using less land directly compared with 2004. At the regional level, the largest expansion in cropland occurred in sub-Saharan Africa (by 15.7 million hectares), and the largest reduction was observed in the US (by 10.5 million hectares).

*Harvested area* At the global scale, harvested area increased by 94 million hectares between 2004 and 2011. As mentioned earlier in this paper, the area of cropland has increased by 17.5 million hectares during the same time period. Comparing these two figures indicates that the harvested area has grown faster than land cover between 2004 and 2011. This could be due to some combination of reductions in crop failure and idled land and increases in double cropping between 2004 and 2011. The largest expansions in harvested area occurred in sub-Saharan Africa (by 32.5 million hectares), India (by 21.9 million hectares), and China (by 13.7 million hectares). Harvested area decreased in a few regions slightly.

Among crops at the global scale, the largest expansion in harvested area is for oilseeds (by 33.2 million hectares). At the global scale, the smallest increase in harvested area was for wheat. The harvested area of wheat increased only by 3.4 million hectares between 2004 and 2011.

Harvested area decreased in all crop categories in the US, except for coarse grains. The harvested area of coarse grains increased by 2 million hectares. This reflects the need for more corn for ethanol production in the US. In the EU, the harvested area of almost all crops decreased, except for oilseeds. This reflects the need for more oilseeds for biodiesel production in the EU.

*Crop production* At the global level, production of paddy rice, wheat, coarse grains, oilseeds, and other crops increased by 115.4 million metric tons (MMT), 66.8, 127.7, 178, and 907.3 MMT, repressively, between 2004 and 2011. The per capita production for all of these crop categories also increased by 9, 1.8, 5.5, 18.7, and 52 kg, respectively. Thus, more food is available to consume per person. Of course, some of these crops are consumed for non-food uses (e.g., corn for ethanol or oilseeds for biodiesel), but some of them (like rice and wheat) are basic food crops.

The largest increases in crop production occurred in Brazil (by 368.6 MMT), China (by 325.7 MMT), India (by 305.9 MMT), and sub-Saharan Africa (by 128.2 MMT) between 2004 and 2011. Crop production has fallen (by 68.4 MMT) in Canada. Again, that is basically due to a correction in the GTAP data for Canada as indicated above. In the US only production of coarse grains has increased by 4.2 MMT, while production of other crops has decreased between 2004 and 2011.

*Yield* Crop yields increased in many regions. At the global scale rice, wheat, coarse grains, oilseeds, and other crop yields increased by 9.7, 8.8, 7.8, 13.8, and 7.2%, respectively, between 2004 and 2011. The largest growth in crop yields occurred in Brazil (ranging from 26 to 38%), India (ranging from 10 to 40%), Russia (ranging from 10 to 35%), and members of the former Soviet Union (ranging from 15 to 40%). In many other regions, yields also increased by large percentages.

In the US, yield has slightly increased for paddy rice, wheat, and other crops, and decreased for coarse grains (by 4%) and soybeans (by 0.2%) between 2004 and 2011. It is important to note that the US corn yield was more than 10 metric tons per hectare in 2004, higher than the normal trend. On the other hand, it was about 9.2 metric tons per hectare in 2011, below the normal trend.^1^ Therefore, while corn yield follows an upward trend in the US, our data show a reduction in coarse grain yield between 2004 and 2011.

*Cropland pasture* Cropland pasture represents a portion of cropland which has been cultivated and used for crop production in the past, but currently is in pasture. The GTAP-BIO 2004 database includes cropland pasture only for US (25 million hectares) and Brazil (23.6 million hectares). The area of cropland pasture in US has dropped to 5.2 million hectares in 2011, according to the US census. Due to the lack of information, we assumed that the area of cropland pasture in Brazil has dropped to 11.8 million hectares in 2011. Finally, with access to new data, about 5.2 million hectares of cropland pasture was added to the database for Canada.

### Improvements in GTAP-BIO model

Birur et al. [1] used an improved version of the GTAP-E model [41] and developed the first version of the GTAP-BIO model to analyze the impacts of biofuel production on energy and agricultural markets and to study the market. This early model version was able to trace market-mediated responses due to biofuel production. Responses such as but not limited to: (1) increases in crop prices due to expansion in feedstock demand for biofuel production; (2) reductions in crop demands in non-biofuel uses such as food and feed; (3) changes in the global trade of crops and other agricultural products; (4) expansion in crop supplies across the world; (5) substitution between biofuels and fossil fuels; (6) crop switching as relative prices changed; and (7) competition for limited resources. However, the model was not able to accurately quantify these impacts and was missing several other important market-mediated responses due to several limitations.

The first version of the model did not include biofuel by-products such as Distiller’s Dried Grains with Soluble (DDGS) and oilseed meals. Hence, the model was missing the impacts of biofuel production on the livestock industry and animal feed rations. Therefore, it provided misleading results on livestock demand for crops, leading to overestimation of biofuel impacts on demand for crops and land use changes. In addition, the first model did not consider the fact that productivity of new land likely would be lower than the existing cropland. Furthermore, the first model did not include any yield response to higher crop prices. More importantly, it was incapable to trace changes in physical land. Over the past decade, many modifications were introduced to GTAP-BIO to improve its performance and eliminate its initial deficiencies. Golub and Hertel [42] explained some of the early modifications. Here, we briefly outline them and introduce some newer modifications.

Taheripour et al. [3, 4] introduced biofuel by-products in the model and defined a module to take into account substitution between biofuels by-products (such as DDGS and oilseed meals) and feed crops in livestock feed rations. Hertel et al. [20] improved the model to distinguish between productivities of the new and existing croplands. They developed a new land supply system to trace changes in physical land. In addition, they defined a module to better take care of crop yield responses to changes in crop prices and production costs. The impacts of these modifications on the outcomes of the model were substantial, basically leading to lower induced land use changes compared with the initial model.

The three main modifications made by Hertel et al. [20] were significant contributions. However, these authors established their modifications based on some limited real-world observations. First, they assumed that the productivity of new land is about 2/3 of the productivity of existing cropland everywhere across the world. Second, they assumed that the land transformation elasticity among forest, pasture, and cropland equals to 0.2 across the world, and also used a uniform land transformation elasticity of 0.5 to govern allocation of cropland across alternative crops everywhere around the world. Finally, they assumed that crop yield response with respect to changes in profitability of crop production is uniform across regions and crops. They also assumed that crop harvest frequency remains fixed, meaning no expansion in multiple cropping and no conversion of idled cropland to crop production. Many of these limitations were removed over time.

Tyner et al. [23] partially removed the last issue mentioned above by introducing cropland pasture into the model for only US and Brazil, where data were available. Cropland pasture is a particular marginal cropland that usually is used as pasture land but moves to cropland when more cropland is needed. The model developed by these authors and the subsequent work continued to ignore multiple cropping and assumed idled cropland will remain idle.

Taheripour et al. [5] used a biophysical model (TEM) and estimated a set of extensification parameters which represent productivity of new cropland versus the existing land by region at the spatial resolution of Agro-Ecological Zone. Using a tuning process, Taheripour and Tyner [29] developed a set of land transformation elasticities by region according to recent real-world observations on land use changes across the world. These land transformations elasticities govern land allocation across land cover categories and distribute cropland among crops.

Recently, Taheripour et al. [43] introduced several more important improvements: First, they altered the land use module of the model to take into account intensification in cropland due to multiple cropping and/or returning idled cropland to crop production. They defined a new set of regional intensification parameters and determined their magnitudes according to observed land use changes across the world in recent years. They also altered the assumption that the elasticity of yield improvement with respect to changes in profitability of crops is uniform across regions. Instead, they defined regional yield responses and tuned their magnitudes according to observed regional changes in crop yields.

These model improvements were targeted towards the first-generation biofuels. Taheripour and Tyner [44] developed a special version of the model (called GTAP-BIO-ADV) to examine the economic and land use impacts of the second-generation biofuels. Unlike other versions of the GTAP-BIO model which put all crops in one nest in the land supply tree, the GTAP-BIO-ADV model uses a different land supply tree which puts cropland pasture and dedicated crops (such as miscanthus and switchgrass) in one nest and all other crops in another nest and allows the land to move between the two nests. They used this setup to avoid conversion of food crops to dedicated energy crops to make greater use of cropland pasture (a representative for marginal land) to produce dedicated energy crops. The GTAP-BIO-ADV model was developed prior to the tuning process described above and only includes those model modifications that were available when the model was developed in 2011.

This paper brings all the modifications explained above less than one umbrella and generates a comprehensive model to have the first- and second-generation biofuels in one model. We also match the model with the 2011 GTAP-BIO database introduced in the data section. Then, we examined the land use impacts and the biofuel pathways outlined in the next sections. Henceforth, we refer to this model as GTAP-BIO-ADV11.

**Fig. 1:**
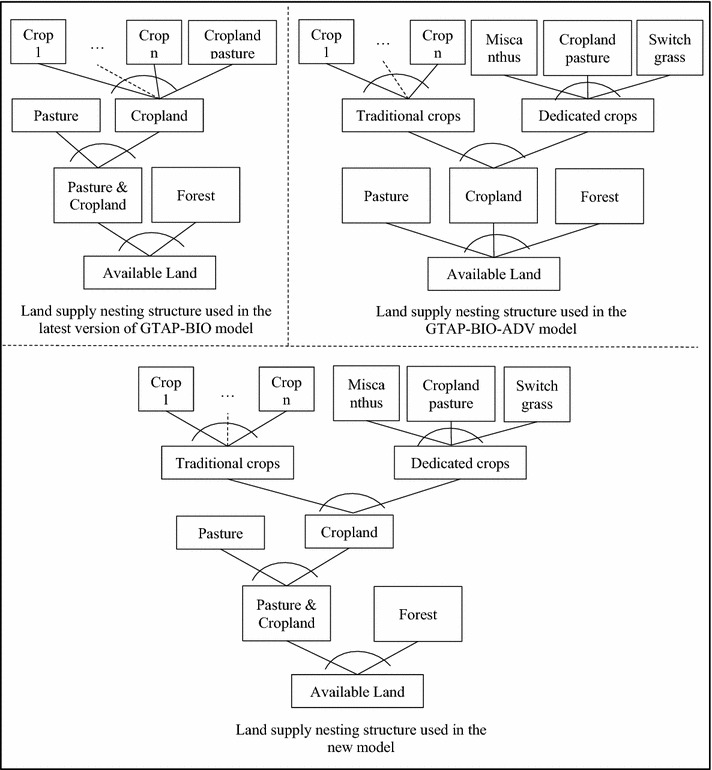
Land supply trees in alternative versions of GTAP-BIO model

The modeling framework used in this paper is based on the latest model introduced by Taheripour et al. [43] which includes all the modification made in the GTAP-BIO model over time including intensification in cropland due to multiple cropping and returning idled cropland to crop production. To do simulations for the second-generation biofuels, we alter the land supply tree of this model according to the land supply tree of the GTAP-BIO-ADV model. The top left and right panels of Fig. 1 represent the land supply trees of the latest version of the GTAP-BIO and GTAP-BIO-ADV models, respectively. The bottom panel of this figure shows the mix of these two panels which we used in this paper. As shown in the bottom panel, the land supply tree of the new model uses two nests to govern changes in land cover and two nests to manage allocation of cropland among crops, including miscanthus and switchgrass. At the lowest level of this tree, available land is allocated between forest and a mix of cropland–pasture. The second level allocates the mix of cropland–pasture to cropland and pasture. Then, at the third level, cropland is divided between the traditional crops (first nest of cropland) and dedicated crops including cropland pasture (second nest of cropland). Finally, at the top level, the first category of land is allocated among the traditional crops, and the second category between miscanthus, switchgrass, and cropland pasture.

The land transformation elasticities used with this specification match the tuned elasticities reported by Taheripour and Tyner [29] for the land cover and allocation of cropland among the traditional crops. For the cropland nest including miscanthus, switchgrass, and cropland pasture, following Taheripour and Tyner [44], we used a relatively large land transformation elasticity to support the idea of producing dedicated crops on marginal cropland and to avoid a major competition between the traditional crops and dedicated energy crops. For the nest between the first and second groups of cropland, we use the same tuned land transformation elasticities which we used in land allocation among the first group of crops (i.e., traditional crops). With this assignment, the new model replicates the results of the old model for the first-generation biofuels.

The modeling framework developed by Taheripour et al. [43] takes into account intensification in cropland due to multiple cropping and/or conversion of unused cropland. These authors introduced a new land intensification factor into the model and tuned it according to the actual recent historical observations. The modeling framework used in this paper adopts the approach developed by these authors. However, it required changes to introduce land intensification in the new model which uses a different land supply structure.

With a one-nest cropland structure used by Taheripour et al. [43], the relationship between changes in harvested area and changes in cropland in the presence of land intensification can be captured by the following equation^2^:

1 $$h_{j} = {\text{tl}} + \theta \left[ {{\text{pl}} - {\text{ph}}_{j} } \right].$$

Here, tl = *l* + afs, *h*_*j*_ represents changes in the harvested area of crop *j*, *l* indicates changes in available cropland due to deforestation (conversion from forest or pasture to cropland and vice versa), afs stands for changes in available land due to intensification (shift factor in land supply), *θ* shows the land transformation elasticity which governs allocation of land among crops, pl demonstrates changes in the cropland rent, and finally, ph_*j*_ denotes changes in the land rent for crop *j*.

With a two-nest cropland nesting structure, presented in the bottom panel of Fig. 1, the following four relationships establish the links between changes in cropland and harvested areas in the presence of land intensification:

2 $$l_{1} = {\text{tl}} + \emptyset \left[ {{\text{pl}} - {\text{ph}}_{1} } \right],$$

3 $$l_{2} = {\text{tl}} + \emptyset \left[ {{\text{pl}} - {\text{ph}}_{2} } \right],$$

4 $$h_{1j} = l_{1} + \omega_{1} \left[ {{\text{pl}}_{1} - {\text{ph}}_{1j} } \right],$$

5 $$h_{2j} = l_{2} + \omega_{2} \left[ {{\text{pl}}_{2} - {\text{ph}}_{2j} } \right].$$

In these equations, *tl*, ${\text{afs}}$, and pl carry the same definitions as described above. Other variables are defined as follows:

- *l*_1_ and *l*_2_ represent changes in the first and second branches of cropland.
- ph_1_ and ph_2_ indicate changes in the rents associated with the first and second branches of cropland.
- *h*_1*j*_ and *h*_2*j*_ stand for changes in the harvested areas of crops included in the first and second groups of crops.
- ph_1*j*_ and ph_2*j*_ show changes in the rents associated with each crop included in the first and second groups of crops.
- ∅ demonstrates the land transformation elasticity which governs allocation of cropland among the first and second groups of crops.
- *ω*_1_ shows the land transformation elasticity which governs allocation of the first branch of cropland among the first group of crops; and finally.
- *ω*_2_ represents the land transformation elasticity which governs allocation of the second branch of cropland among the second group of crops.

**Fig. 2:**
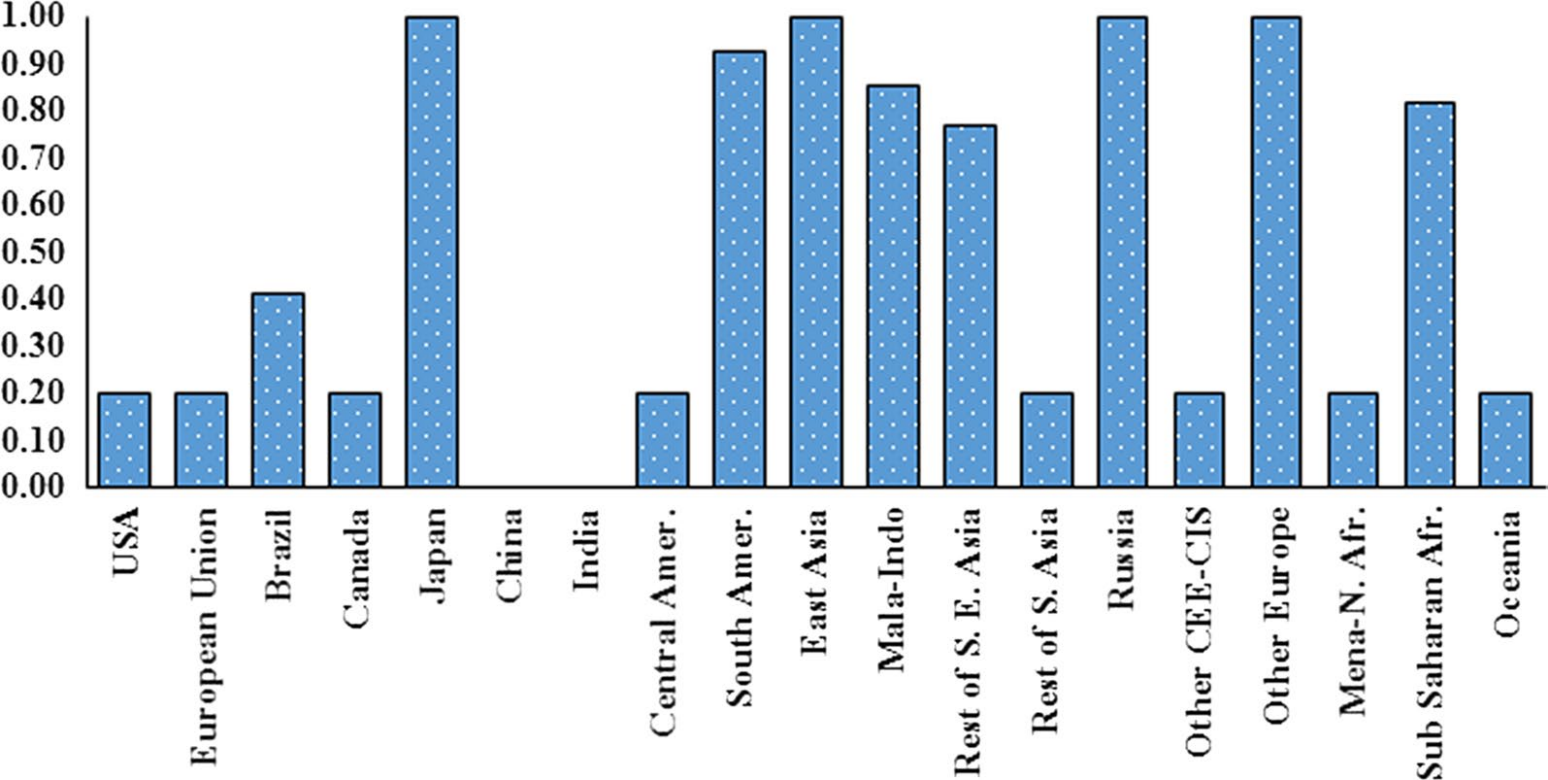
Tuned regional land intensification parameters ($\gamma_{\text{r}}$)

Taheripour et al. [36] used several relationships to introduce land intensification (due to multiple cropping and or conversion of unused land to cropland) and endogenously determine the size of afs by region. Among all modifications, they used to accomplish this task, they introduced a parameter, called intensification factor and denoted by *γ*_r_, which represents the magnitude of intensification by region. This parameter varies between 0 and 1 (i.e. 0 ≤ *γ*_r_ ≤ 1). When $\gamma_{\text{r}} = 1,$ there is no land intensification. In this case, any expansion in harvested area leads to an expansion in cropland which comes from conversion of forest and/or pasture. On the other hand, when $\gamma_{\text{r}} = 0,$ it shows that an expansion in harvested area will not expand cropland. In this case, the additional harvested area comes from multiple cropping and/or converting unused cropland to crop production. Taheripour et al. [43] determined the regional values for this parameter, according to recent observed trends in land intensification across the world. Figure 2 represents the regional values of this parameter.

As shown in Fig. 2, in China and India, the parameter of land intensification equals 0, indicating that in these two countries, an expansion in harvested area does not lead to an expansion in cropland. On the other hand, in some countries/regions, the parameter of land intensification is close to 1, for instance Japan and East Asia. In these regions, any expansion in harvested area will equal an identical expansion in cropland with no intensification. Finally, in some countries/regions, the land intensification parameter is in between 0 and 1, say in Brazil and sub-Saharan Africa. In these regions, a portion of expansion in harvested area comes from land intensification and a portion from expansion in cropland. We use these values in our new model with one exception. For the case of Malaysia–Indonesia region, while the intensification parameter is less than 1, we assumed no intensification in this region, because it is the main source of palm oil and multiple cropping for palm tree is meaningless.

**Fig. 3:**
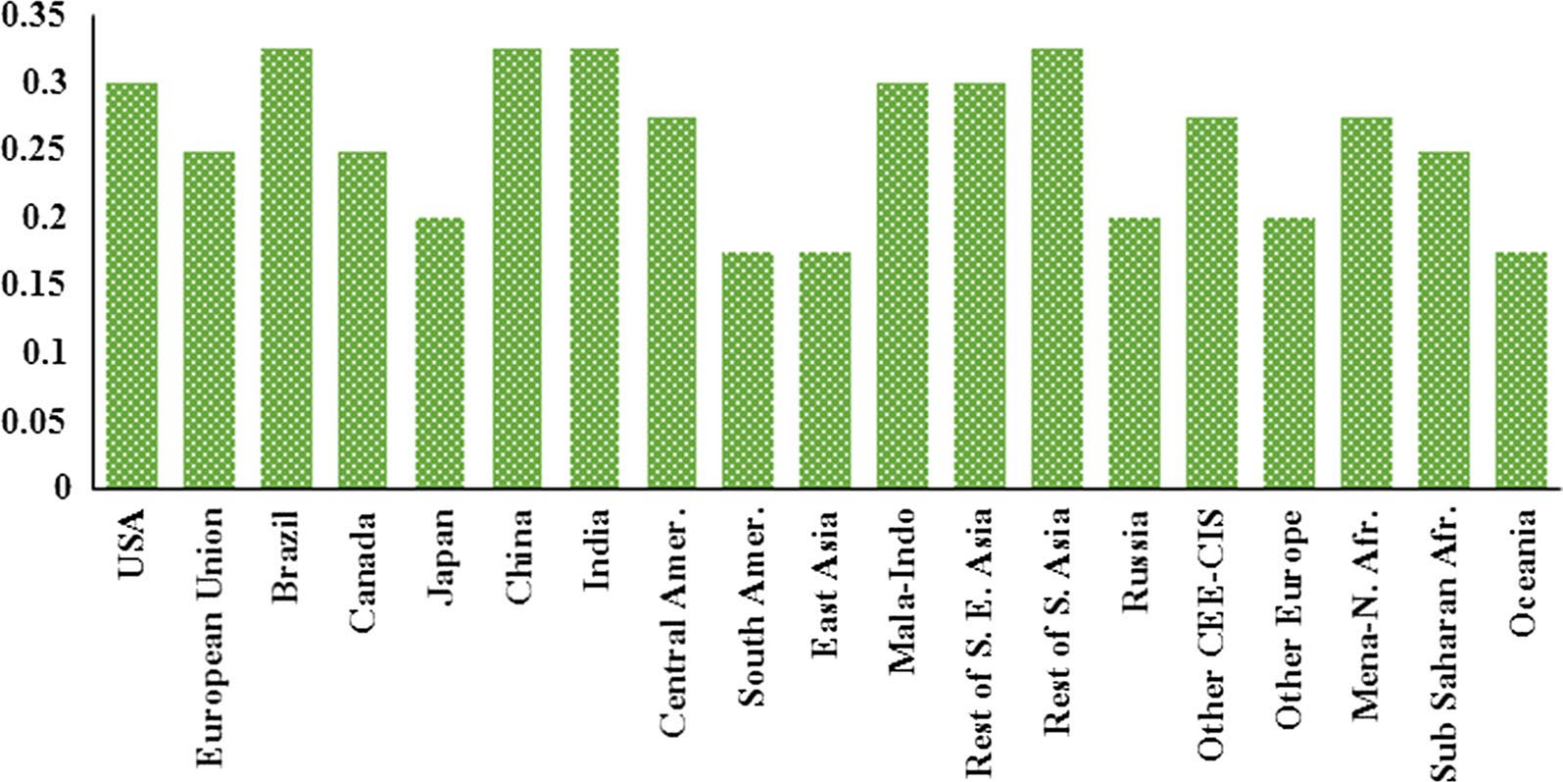
Tuned regional yield to price elasticities (${\text{YDEL}}_{\text{r}}$)

Following the existing literature [45, 46] which confirms yield improvement due to higher crop prices, Taheripour et al. [43] developed a set of regional elasticities which show yield to price response (known as YDEL) by region. Figure 3 represents these regional yield elasticities. Unlike the earlier version of the GTAP-BIO model which commonly assumed YDEL = 0.25, as shown in Fig. 2, the size of this elasticity varies between 0.175 and 0.325. Several regions including South America, East Asia, and Oceania have the lowest yield response, while Brazil has the highest rate.

## Results

We developed several experiments to examine induced land use changes and emissions for the following first- and second-generation biofuel pathways using the GTAP-BIO-ADV11 model:

*Experiment 1: * Expansion in US corn ethanol by 1.07 BGs (from 13.93 BGs in 2011 to 15 BGs);
*Experiment 2: * Expansion in US soybean biodiesel by 0.5 BGs;
*Experiment 3: * Expansion in US miscanthus bio-gasoline by 1 BGs.

The bio-gasoline produced in the third experiment contains 50% more energy compared to corn ethanol. Since producing biofuels from agricultural residue (e.g., corn stover) does not generate noticeable land use changes [44], we did not examine ILUC for these biofuel pathways. We use an improved version of the emissions factor model developed by Plevin et al. [47] to convert the induced land use changes obtained from these simulations to calculate the induced land use emissions for each biofuel pathway. The earlier version of this model was not providing land use emission factors for converting land to dedicated energy crops such as miscanthus and switchgrass. Several papers have shown that producing dedicated energy crops on marginal lands will increase their carbon sequestration capabilities and that helps to sequester more carbon in marginal lands (for example, see [45]). The new emissions factor model provides land use emission factor for converting land to dedicated energy crops and takes into account gains in carbon stocks due to this conversion. The data for calibration of the new component in AEZ-EF were taken from the CCLUB model provided by Argonne National Laboratory [48]. Finally, it is important to note that the emission factor model takes into account carbon fluxes due to conversion of forest, pasture, and cropland pasture to cropland and the reverse.

### Land use changes

The induced land use changes obtained from the examined biofuel pathways are presented in Table 1. The expansion in US ethanol production from its 2011 to 15 BGs increases the global harvested area of corn by about 621 thousand hectares, after taking into the expansion in DDGS in conjunction with ethanol production. The expansion in demand for corn encourages farmers to switch from other crops (e.g., wheat, soybeans, and several animal feed crops) to corn due to market-mediated responses. That transfers a net of 349 thousand hectares from other crops to corn at the global scale. In addition, the area of cropland pasture (a marginal land used by livestock industry) drops by 129 thousand hectares in the US, Brazil, and Canada. Hence, about 478 (i.e. 349 + 129) thousand hectares of the land requirement for corn production comes from reductions in other crops and cropland pasture. Therefore, at the end, harvested area increases only by 143 (i.e. 621–478) thousand hectares, as shown in Table 1. However, due to intensification, cropland area grows only by 69.4 thousand hectares. This means that about 51% of the need for expansion in harvested area is expected to be covered by multiple cropping and/or using idled cropland. Therefore, the land requirement for 1000 gallons of corn ethanol is about 0.06 hectares in the presence of land intensification. Ignoring intensification, the land requirement increases to 0.13 hectares per 1000 gallons of ethanol.

**Table 1:** Induced land use changes for alternative biofuel pathways (thousand hectares)

Description	USA	EU	Brazil	Canada	Sub-Saharan Africa	Others	World
Experiment 1 US corn ethanol							
Forest	3.0	−0.9	−3.8	−0.8	−8.8	−10.6	−22.0
Pasture	−7.3	−2.0	−8.1	−0.4	−22.3	−7.5	−47.5
Cropland	4.3	3.0	11.8	1.2	31.0	18.0	69.4
Harvested area	19.4	15.0	25.8	6.2	40.7	35.6	142.6
Cropland pasture	−74.8	0.0	−39.8	−14.1	0.0	0.0	−128.7
Experiment 2 US soybean biodiesel							
Forest	−0.9	−0.4	−2.0	−0.4	3.6	−3.7	−3.8
Pasture	−1.2	−0.8	−3.4	0.0	−16.4	−10.6	−32.5
Cropland	2.0	1.2	5.4	0.4	12.9	14.3	36.2
Harvested area	9.5	5.8	11.7	2.2	16.6	19.1	64.9
Cropland pasture	−42.6	0.0	−18.1	−5.2	0.0	0.0	−66.0
Experiment 3 US miscanthus bio-gasoline							
Forest	−41.0	−1.4	−5.7	−1.7	−13.7	−20.1	−83.7
Pasture	39.3	−2.2	−8.3	0.2	−23.9	−0.9	4.2
Cropland	1.8	3.7	13.9	1.5	37.6	21.0	79.7
Harvested area	9.1	18.2	30.3	7.7	49.6	41.6	156.4
Cropland pasture	−1017.3	0.0	−45.6	−14.7	0.0	0.0	−1077.6

In addition to changes in land cover, expansion in corn ethanol generates changes in the mix of cropland. In particular, it transfers some cropland pasture to the traditional crops. For the expansion in corn ethanol from 2011 to 15 BGs, about 129 thousand hectares of cropland pasture will be converted to the traditional crops, as shown in the first panel of Table 1. This is about 0.12 hectares per 1000 gallons of ethanol. For the case of corn ethanol, deforestation covers 32% of the land requirement and the rest (68%) is due to conversion of pasture to cropland.

An expansion in soybean biodiesel produced in the US by 0.5 BGs increases the global harvested area by about 64.5 thousand hectares, but only 56% of this expansion transfers to new cropland due to intensification. Therefore, global cropland increases by 36.1 thousand hectares. The index of land requirement for 1000 gallon of soybean biodiesel is about 0.07 hectares. Ignoring the land intensification, this index jumps to 0.13 hectares per 1000 gallons of soybean biodiesel. These indices are similar to their corresponding values for the cases of corn ethanol. For this pathway, the rate of conversion from cropland pasture to traditional crops is about 0.13 hectares per 1000 gallons of biodiesel, very similar to the corresponding rate for corn ethanol.

We now turn to induced land use changes for cellulosic biofuels produced from dedicated energy crops such as miscanthus or switchgrass. The narrative of induced land use changes for these biofuels is entirely different from the description of induced land use changes for the first-generation biofuels producing biofuels (say ethanol) from traditional crops (say corn) generates market-mediated responses such as reduction in consumption of crops in non-biofuel uses, switching among crops, expansion in biofuels by-products (which can be used in livestock feed rations instead of crops), and yield improvement. These market-mediated responses reduce the land use impacts of producing biofuels from traditional crops as described by Hertel et al. [20]. However, producing cellulosic biofuels from energy crops such as miscanthus or switchgrass may not generate these market-mediated responses.

For example, consider producing bio-gasoline from miscanthus, which we examine in this paper. This pathway produces no animal feed by-product. Therefore, an expansion in this biofuel does not lead to a reduction in livestock demand for crops. Miscanthus is not used in other industries. Hence, we cannot divert its current uses to biofuel production. Thus, miscanthus should be produced for every drop of bio-gasoline. For example, if we plan to produce 1 BGs of miscanthus bio-gasoline, then we need about 775 thousand hectares of land (with a conversion rate of 66.1 gallons per metric ton of miscanthus and 19.5 metric tons of miscanthus per hectare as we assumed in developing the GTAP-BIO database). Now, the question is: From where will the required land for miscanthus production come?

It is frequently argued that dedicated energy crops should not compete with the traditional food crops. This means no or little conversion from the traditional food-feed crops to cellulosic energy crops. It is also commonly believed that cellulosic energy crops should be produced on low-quality “*marginal land*”. Beside this widespread belief, the definition and availability of “*marginal land*” are subject to debate [49]. If the low-quality marginal land is entirely unused, then producing cellulosic crops on these lands may not significantly affect competition for land. In this case, unused land will be converted to miscanthus as needed to meet the feedstock demand for the stipulated expansion in cellulosic biofuel.

However, if the low-quality marginal land is used by livestock producers as grazing land (e.g., cropland pasture in the US), then producing energy crops on cropland pasture directly and indirectly affects the livestock industry, and that generates some consequences. In this case, the livestock industry demands more feed crops, uses more processed feed, and/or converts natural forest to pasture in response to converting cropland pasture to miscanthus.

Now, consider the induced land use changes for the third experiment which extends production of the US bio-gasoline from miscanthus by 1 BGs. As shown in the bottom panel of Table 1, the anticipated expansion in miscanthus bio-gasoline increases the global harvested area by 156.4 thousand hectares. However, due to intensification, the global cropland area grows only by 79.7 thousand hectares. Therefore, the index of land requirement for 1000 gallons of miscanthus bio-gasoline is about 0.08 hectares in the presence of land intensification. Ignoring intensification, the index of land requirement increases to 0.16 hectares per 1000 gallons of bio-gasoline. These land requirement indices are not very different from the corresponding figures for corn ethanol. However, three is a major difference between corn ethanol and miscanthus bio-gasoline when we compare their impacts on cropland pasture.

As shown in Table 1, an expansion in US miscanthus bio-gasoline by 1 BG converts 1077.6 thousand hectares of cropland pasture to cropland. This is about 1.08 hectares per 1000 gallons of miscanthus bio-gasoline. This figure is approximately 9 times higher than the corresponding figure for corn ethanol. This difference is because producing miscanthus bio-gasoline does not create the market-mediated responses which corn ethanol generates. The change in cropland pasture area (i.e., 1077.6 thousand hectare) is higher than the direct land requirement for producing 1 BG of miscanthus bio-gasoline (i.e., 763 thousand hectares). When the livestock industry gives up cropland pasture at a large scale, it uses more feed crops and/or processed feed items, and that generates some land use changes including more conversion of cropland pasture to traditional crops. Furthermore, a large conversion of cropland pasture to miscanthus increases the rental value of pasture land (a substitute for cropland pasture) significantly, and that generates some incentives for a mild deforestation in the US, as shown in the lowest panel of Table 1. In the third experiment, the price of miscanthus increases by 53% and the livestock price index (excluding non-ruminant) goes up by about 0.5% which is 5 times higher than the corresponding figure for the forestry sector. Pasture rent grows at about 5% across US AEZs, while the corresponding rate for forest is less than 1%. For the case of corn ethanol, which induces mild conversion of cropland pasture forest and pasture rents grow similarly at rates less than 1% across AEZs in the US. Finally, it is important to note that the tuned land transformation elasticity for forest to agricultural land in the US is small, according to recent observations [29]. In conclusion, while producing miscanthus bio-gasoline slightly increases demand for cropland, it induces major shifts in marginal land (say cropland pasture) to miscanthus production.

### Land use emissions

First, consider induced land use emissions for the first-generation biofuels including corn ethanol and soybean biodiesel for four alternative modeling and database cases: (1) 2004 database with no intensification; (2) 2004 database with intensification; (3) 2011 with no intensification; and (4) 2011 with intensification. The emission results for the first three cases (i.e., cases 1, 2, 3) are taken from Taheripour et al.  [43]. The last case represents the results of the simulations conducted in this paper.

**Fig. 4:**
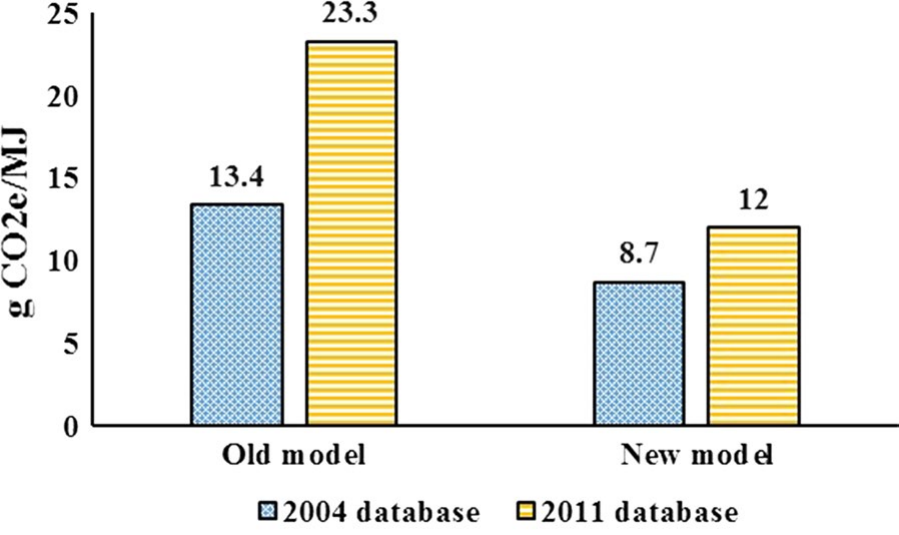
Induced land use emissions for corn ethanol with 2004 and 2011 databases with and without land intensification

Figure 4 shows the results for corn ethanol. With intensification in cropland, an expansion in US ethanol from its 2011 level to 15 BGs generates 12 g CO_2_e/MJ emissions. The corresponding simulation with no intensification generates 23.3 g CO_2_e/MJ emissions. This means that the new model which takes into account intensification in cropland and uses tuned regional YDEL parameters generates significantly lower emissions, approximately by half. The corresponding cases obtained from the 2004 databases represent the same pattern, but demonstrate lower emissions rates. An expansion in corn ethanol from its 2004 level to 15 BGs generates 8.7 g CO_2_e/MJ emissions with intensification and 13.4 g CO_2_e/MJ with no intensification.

These results indicate that the 2011 database generates higher emissions for corn ethanol compared with the 2004 databases, regardless of modeling approach. However, the new model which takes into account intensification in cropland and uses tuned regional YDEL values projects lower emissions, regardless of the implemented database. The 2011 database generates more emissions for corn due to several factors including but not limited to: (1) less availability of cropland pasture in the US in 2011; (2) less flexibility in domestic use of corn in 2011; (3) less flexibility in US corn exports in 2011; (4) smaller US corn yield in 2011; (5) more reductions in US crop exports (in particular soybean and wheat) in 2011; (6) larger DDGS trade share in 2011; (7) smaller capital share in corn ethanol cost structure; and (8) finally, the marginal land use impacts of ethanol in 2011 are much larger than 2004, because the base level of ethanol in 2011 is much larger than 2004.

**Fig. 5:**
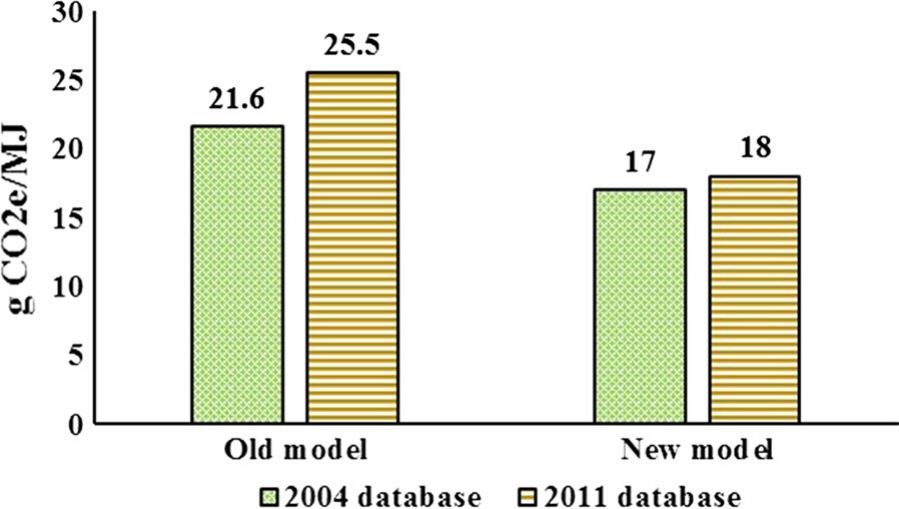
Induced land use emissions for soybean biodiesel with 2004 and 2011 databases with and without land intensification

Figure 5 shows the results for soybean biodiesel. In the presence of intensification in cropland, an expansion in the US soybean biodiesel by 0.5 BGs generates 18 g CO_2_e/MJ emissions. The corresponding simulation with no intensification generates 25.5 g CO_2_e/MJ emissions. This means that, similar to the cases for corn ethanol, the new model which takes into account intensification in cropland and uses tuned regional YDEL parameters generates significantly lower emissions. The corresponding cases obtained from the 2004 databases represent the same pattern. An expansion in the US soybean biodiesel by 0.5 BGs generates 17 g CO_2_e/MJ emissions with intensification and 21.6 g CO_2_e/MJ with no intensification. Furthermore, producing soybean biodiesel in the US encourages expansion in vegetable oils produced in some other countries including more production of palm oil in Malaysia and Indonesia on peat land, which entails extremely high emissions. This is one reason why land use change emissions induced by US soybean biodiesel production are generally higher than those induced by US corn ethanol production.

Unlike the case of corn ethanol, these results indicate that the 2011 database generates slightly higher emissions for soybean biodiesel compared with the 2004 databases, regardless of modeling approach. This observation is due to several factors including but not limited to: (1) conversion of a larger portion of US soybean exports to domestic use in 2011 which reduces the size of land conversion in US; (2) Brazil, Canada, and other countries produce more soybeans in 2011; (3) significantly larger oilseed yields across the world (except for US) generates weaker land conversion outside the US; (4) larger availability of oilseed meals in 2011 which contributes to a higher share of pasture in 2011; and larger share of palm oil in total vegetable oils in 2011.

We now turn to induced land use emissions for miscanthus bio-gasoline. Two alternative cases are examined to highlight the role of soil carbon sequestration gained from production of miscanthus on marginal land. First, we assume that producing miscanthus on cropland pasture does not improve soil carbon sequestration. Then, following the literature [48, 49]^3^, we take into account the fact that producing miscanthus on marginal land improves the soil carbon content. The existing literature confirms that producing miscanthus on marginal land improves its soil carbon content.

**Fig. 6:**
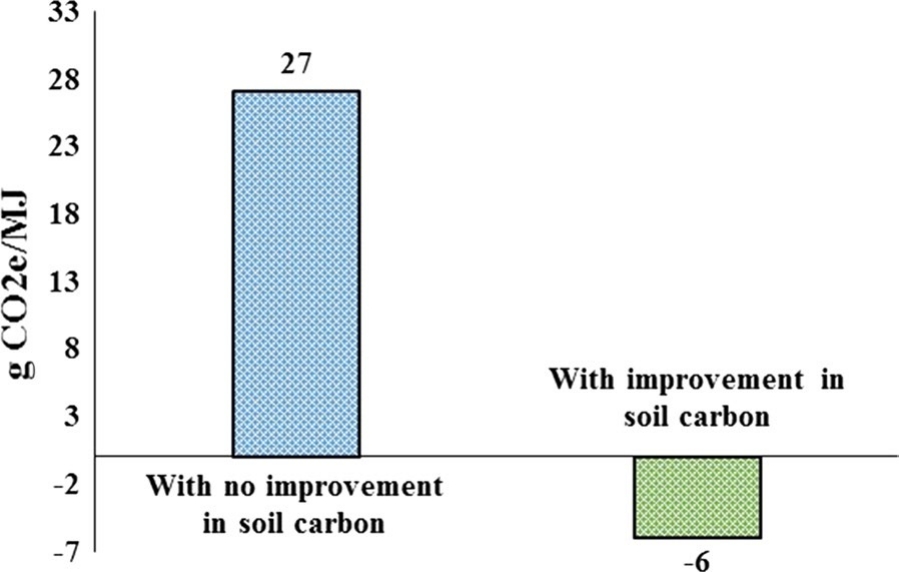
Induced land use emissions for miscanthus bio-gasoline with and without including improvements in soil carbon sequestration

For the first case, an expansion in US miscanthus bio-gasoline by 1 BGs generates about 27 g CO_2_e/MJ emissions. Compared with corn ethanol and soybean biodiesel, this figure is large. As mentioned before, an expansion in US miscanthus bio-gasoline by 1 BGs transfers about 1117.6 thousand hectares of cropland pasture to miscanthus production and other tradition crops. Only about 70% of this conversion goes to miscanthus. Hence, if we ignore the carbon saving from miscanthus production, then producing bio-gasoline from miscanthus generates more emissions than corn ethanol. For the second case, as shown in Fig. 6, the emissions score for miscanthus to bio-gasoline drops to about −6 g CO_2_e/MJ. This figure is in line with the results reported by Wang et al. [50]. These authors used induced land use results obtained from an earlier version of the GTAP model and emissions factors from the CCLUB calculated that producing ethanol from miscanthus generates negative land use emissions by −7 g CO_2_e/MJ. On the other hand, Dwivedi et al. [45], who used farm and firm level data in combination with some limited field experiments, reported that converting miscanthus to ethanol generates about −34 to −59 g CO_2_e/MJ land use emissions. These results underscore the fact that for the case of cellulosic biofuels, the magnitude of induced land use emissions varies significantly by the method of calculating land use changes and largely depends on the assigned emission factor to the converted marginal land.

## Conclusions

In this paper, we have covered three major modifications to the GTAP-BIO model. First, we reviewed the change from using the 2004 database to 2011. Many changes in the global economy occurred between 2004 and 2011 including the development of the first-generation biofuels in many world regions, changes in crop production area and yields, and vast changes in the levels and mix of GDP in many world regions. All these changes and many others have a profound impact on any simulations that are performed using the 2011 database versus the older 2004 data. Of course, moving forward, we must use the updated data, so it is important to understand the significance of the major changes, particularly as they impact biofuels and land use.

The second major change was a revision of the GTAP-BIO model to better handle intensification. The previous versions of the GTAP model and other similar models assumed that a change in harvested area equals a change in land cover. Examining the FAO data, it was clear that this is not the case, so we used that data to develop and parameterize differences in changes at the intensive and extensive margins for each world region. We also calibrated the yield price elasticity by region, as the FAO data also indicated significant differences in yield response by region.

The third major change was to develop a new version of the model (GTAP-BIO-ADV11) used to evaluate land use changes and emissions for dedicated cellulosic feedstocks such as miscanthus. These dedicated energy crops are not similar to the first-generation feedstocks in the sense that they do not generate the level of market-mediated responses we have seen in the first-generation feedstocks. The major market-mediated responses are reduced consumption, crop switching, changes in trade, changes in intensification, and forest or pasture conversion. There is no current consumption or trade in miscanthus. There are no close crop substitutes. Most of the land needed for miscanthus production comes from cropland pasture. Since that is an input into livestock production, more land is needed to produce the needed livestock inputs (which is a market-mediated response). Thus, miscanthus (and other similar cellulosic feedstocks) will need more land that required to actually grow the feedstock. Then, the emissions for the cellulosic feedstocks depend on what we assume in the emissions factor model regarding soil carbon gained or lost in converting land to miscanthus. Much of the literature suggests miscanthus actually sequesters carbon, when grown on the existing cropland or even marginal land. When we take into account this important fact, land use change emissions due to production of bio-gasoline from miscanthus drop to a negative number.

Finally, it is important to note the importance of the new results for the regulatory process. The current CARB carbon scores for corn ethanol and soy biodiesel are 19.8 and 29.1, respectively. The new model and database scores are 12 and 18, respectively, for corn ethanol and soy biodiesel. Thus, the current estimate values are substantially less than the values currently being used for regulatory purposes.

## Abbreviations

GTAP: Global Trade Analysis Project
GHG: greenhouse gas
FAO: Food and Agricultural Organization
CARB: California Air Resources Board
ILUC: induced land use change
LCA: life cycle analysis
EIA: Energy Information Administration
FAOSTAT: FAO Statistics Database
gro: coarse grains (in GTAP)
osd: oilseeds (in GTAP)
vol: vegetable oils and fats (in GTAP)
ofd: food (in GTAP)
BG: billion gallons
GDP: gross domestic product
EU: European Union
MMT: million metric tons
DDGS: distillers dried grains with solubles
US: United States
TEM: Terrestrial Ecosystem Model

## Footnotes

1 The US corn yields for 2004 and 2011 are obtained from the USDA database.

2 This equation only shows the impacts of the shift factor on harvested area. This shift factor appears in several equations of the land supply module. For details, see Taheripour et al. [36].

3 The authors are grateful to Argonne National Laboratory for providing data on carbon sequestration for cellulosic feedstocks and to Dr. Richard Plevin for his work in revising the CARB Agro-ecological Zone Emission Factor (AEZ-EF) Model to handle cellulosic feedstocks.
